# Association of Social Determinants of Health with Acute TBI Care Among U.S. Older Adults

**DOI:** 10.1002/alz.095734

**Published:** 2025-01-09

**Authors:** Catherine Sumida, L. Grisell Diaz‐Ramirez, Kristine Yaffe, W John Boscardin, Raquel C. Gardner, Erica S Kornblith

**Affiliations:** ^1^ San Francisco Veterans Affairs Health Care System, San Francisco, CA USA; ^2^ University of California, San Francisco, San Francisco, CA USA

## Abstract

**Background:**

Older adults have the highest incidence of traumatic brain injury (I‐TBI), which is associated with increased Alzheimer’s disease and related dementia (ADRD) risk. Timely medical assessment, including computed tomography (CT) or magnetic resonance imaging (MRI), is vital for optimal I‐TBI care and recovery. The current study builds on our recent work by examining social determinants of comprehensiveness of I‐TBI care amongst older adults.

**Method:**

Enrollees 65+ years in the nationally representative Health and Retirement Study (HRS), whose HRS survey and Medicare claims data were linked and first TBI diagnosis code occurred between 2000‐2018, were studied. I‐TBI was defined by International Classification of Disease (ICD9&10) inpatient and outpatient codes in Medicare claims (2000‐2018). HRS participants’ acute TBI level of care (TBI‐LC) was ranked from least to most comprehensive: a)neither ER nor imaging codes, b)ER code only, or c)ER and imaging (MRI/CT) codes within ±1day of I‐TBI code. Cognitive status was defined at baseline using Langa‐Weir Dementia Probability, an HRS‐derived predictive model.

**Result:**

Of those respondents with I‐TBI (N = 1148), 15.5% had neither ER nor imaging codes, 14.7% had ER code only, and 69.8% had an ER and imaging code within ±1 day of TBI code. TBI‐LC was associated with rural status (Rao‐Scottc^2^(2) = 11.97, *p* = .003): Rural elders received ER services (20.3% no ER code; 64.9% ER and imaging codes) less frequently compared to non‐rural elders (13.2% no ER code; 72.1% ER and imaging codes). Frequency of ER presentation without imaging were similar amongst rural (14.8%) and non‐rural elders (14.6%). TBI‐LC was not associated with marriage status, income, or cognitive status.

**Conclusion:**

Consistent with prior findings amongst rural adults of all ages, rurality is associated with disparity in acute TBI care comprehensiveness amongst older adults specifically. Interestingly, monthly income, thought to account in part for differences between rural and non‐rural populations, was not associated with TBI care comprehensiveness. These findings suggest further research is needed to examine unique factors contributing to rurality as a barrier to care in elders with TBI (e.g. lack of transportation) and resulting downstream health outcomes (e.g., risk of cognitive decline, ADRD).

